# Awareness regarding Menstrual Hygiene among Girl Students of a School in Chitwan

**DOI:** 10.31729/jnma.4760

**Published:** 2019-12-31

**Authors:** Krishna Bahadur Raut, Roshani Agrawal Khatry, Tumla Shrestha

**Affiliations:** 1 Department of General Practice and Emergency, Chitwan Medical College, Bharatpur, Chitwan, Nepal; 2 Department of Nursing, Patan Academy of Health Sciences, Lalitpur, Nepal; 3 Department of Nursing, Maharajgunj Nursing campus, Maharajgunj, Kathmandu, Nepal

**Keywords:** *adolescent*, *awareness*, *hygiene*, *menstruation*

## Abstract

**Introduction:**

Adolescent girls often lack knowledge regarding reproductive health including menstruation which can be due to socio-cultural barriers in which they grow up. It is important to educate adolescents about issues related to menstruation so that they can safeguard themselves and hold implications for professionals involved in improvement of reproductive health. The main objective of the study was to find out awareness regarding menstrual hygiene among girl students of a school in Chitwan.

**Methods:**

A descriptive cross-sectional study was conducted in a school in Chitwan among girl students of school from grade 8, 9 and 10 from 2019 July to August 2019 after ethical approval. All the girl students from grade 8, 9 and 10 were included into the study. Data were entered and calculations were using Statistical Package for the Social Sciences version 20.

**Results:**

Among 184 adolescent girls, 156 (84.8%) were aware about the menstrual hygiene and most of them 176 (95.7%) knew about the criteria of ideal absorbent to be used during menstruation. Likewise, 120 (65.2%) were aware regarding washing the genital organs, 137 (74.5%) were changing sanitary pad and 136 (73.9%) were disposing used sanitary pad. The mean age of girl students was 14.48±1.259 years respectively.

**Conclusions:**

Awareness regarding menstrual hygiene was present among the girl students, but practice for proper menustral hygiene was low compared to studies done in similar settings.

## INTRODUCTION

Menstruation is the periodic discharge of blood and mucosal tissue from the uterus and vagina.^1^

Different studies in Tanzania and Nigeria has shown most of the women use toilet tissue or cloth instead of sanitary pads.^2,3^ Between 31% and 56% of Nigerian school girls have been found as using toilet tissue or cloth to absorb their menstrual blood as opposed to menstrual pads.^3^ Nearly half of the adolescent girls had not known of the origin of menstrual and had unhygienic practices and misconceptions.^4^

Women spend around six to seven years of their lives menstruating as a result key priority should be given for the necessary knowledge, facilities and cultural environment to manage menstruation hygienically and with dignity for women and girls.In reality, the importance of hygiene during menstrual period is highly neglected.^5^

The main objective of the study was to find out awareness regarding menstrual hygiene among girl students of a school in Chitwan.

## METHODS

A descriptive cross-sectional study design was done at Shree Narayani Vidyamandir Madhyamik Vidyalaya, Shivanagar, Chitwan, Nepal from 2019 July to August 2019 after ethical approval. Data was collected continuously during the study period. Whole population of girl students from grade 8 to 10 were taken into study who already had their menarche. Self-administered structured questionnaire was used to collect data collection within adolescent students of class 8, 9 and 10. Total Sample size was 184 who had already menarche.

Verbal and written consent were also taken from the respondents during this study. The collected data was checked, reviewed and organized for completeness. Data was entered and calculations for binary data was done along with frequency and proportion using SPSS version 20.

## RESULTS

Most 156 (84.8%) of the respondents were aware about the menstrual hygiene and most of them 176 (95.7%) knew about the criteria of ideal absorbent to be used during menstruation. But it was found that they were aware least 2 (1.1%) about how often the absorbent be changed. The respondents had the better awareness regarding others factors like washing the genital organs 120 (65.2%), changing of sanitary pad 137 (74.5%), disposing of sanitary pad 136 (73.9%) and washing of genital organs during menstrual period 165 (89.7%) (Table 1).

**Table 1:** Awareness of therespondents related tomenstrual hygiene

Particulars	n (%)
Meaning of menstrual hygiene	
Aware	156 (84.8)
Unaware	28 (15.2)
Ideally used as absorbent during menstruation	
Aware	176 (95.7)
Unaware	8 (4.3)
Pad/Cloth pad changed during menstruation	
Aware	2 (1.1)
Unaware	182 (98.9)
Cleaning the cloth pad after use	
Aware	161 (87.5)
Unaware	23 (12.5)
Washing the genital organs	
Aware	120 (65.2)
Unaware	64 (34.8)
Changing Sanitary pad how should we decide to wipe the genital	
Aware	137 (74.5)
Unaware	47 (25.5)
Disposing the sanitary pad	
Aware	136 (73.9)
Unaware	48 (26.1)
Washing genital organ 1 1	
Aware	165 (89.7)
Unaware	19 (10.3)

Majority 179 (97.3%) of the total respondents were aware what the menstruation is but only 112 (60.9%) knew the causes. Majority 180 (97.8%) of them aware about the period of menstrual blood flow, organ from which menstrual bleeding occurs 177 (96.2%) but majority 167 (90.8%) of them were unaware about the meaning of menstrual cycle, average cycle of menstruation 46 (25%) and amount of blood flow during this period 112 (60.9%) (Table 2).

**Table 2:** Awareness of the respondents related to menstruation

Particulars	n (%)
Definition of menstruation	
Aware	179 (97.3)
Unaware	5 (2.7)
Causes of menstruation	
Aware	112 (60.9)
Unaware	72 (39.1)
Meaning of menstrual cycle	
Aware	17 (9.2)
Unaware	167 (90.8)
Average cycle of menstruation	
Aware	46 (25.0)
Unaware	138 (75.0)
Period of menstrual blood flow occur in normal menstrual cycle	
Aware	180 (97.8)
Unaware	4 (2.2)
Amount of blood flow during period	
Aware	72 (39.1)
Unaware	112 (60.9)
Organs from which menstrual bleeding occurs	
Aware	177 (96.2)
Unaware	7 (3.8)

The mean age of the respondent was 14.48±1.259 years. Most 92 (50%) of the respondents were of the age group 15 to 17 years and least 1 (0.5%) belonged to age group of 18 to 20 years. Most 86 (46.7%) of respondents belonged to Janajati ethnicity, about two third 145 (78.8 %) were Hindus (Table 3).

**Table 3:** Distribution of socio-demographic variables.

Variables		n (%)
Age of the respondents (in years)		
S.N.	Age Group	n (%)
1.	12-14	91 (49.5)
2.	15-17	92 (50.0)
3.	18-20	1 (0.5)
	Mean age±SD	14.48±1.259
Ethnicity of respondent		
S.N.	Age Group	n (%)
1.	Brahmin	40 (21.7)
2.	Chhetri	31 (16.8)
3.	Janjati	86 (46.7)
4.	Dalit	27 (14.7)
Religion of respondent		
S.N.	Age Group	n (%)
1.	Hinduism	145 (78.8)
2.	Non-Hinduism	39 (21.2)
Grade of respondent		
S.N.	Age Group	n (%)
1.	8^th^ grade	64 (34.8)
2.	9^th^ grade	70 (38.0)
3.	10^th^ grade	50 (27.2)

## DISCUSSION

The finding of the study shows that 97.3% of the total respondents were aware what the menstruation is but only 60.9% knew the causes. One of studies showed that only 31% believed that menstruation was a normal physiological process,^6^ that very findings are contraindicated by the present study. Similarly, this result was contraindicated by another study which showed that only 36.7% of girls knew that it is caused by hormones, 6.0% of girls knew that menstruation is a normal physiological process.^7^

The present study shows that 84.8% of the respondents knew that menstrual hygiene is maintaining proper perineal hygiene during menstruation which is similar to the study which showed that 81% respondents knew about meaning of menstrual hygiene.^8^

The present study findings show that 97.8% of the respondents have the knowledge that period of menstrual blood flow is 3-7 days. Similarly, a study showed that 79 % respondents answered that period of menstrual blood flow is 3-7 days.^9^ This finding is also consistent with another study, where 83.3% respondents answered that period of menstrual blood flow is 3-7 days.^10^

Recent study indicates that 98.9% respondents have no knowledge that to avoid infection and odour, pad should be changed every 4 hours per day. Similar study showed that only 1.1% of respondents were aware that to avoid infection and odour pad should be changed every 4 hours per day.^11^

The findings of the study show that 95.7% respondents opined that new clean cloth and sanitary pad is the ideal absorbent to be used during menstruation. This finding is also similar with study findings, where 97% women opined that sanitary pad is the ideal absorbent to be used during menstruation.^10^

## CONCLUSIONS

Awareness regarding menstrual hygiene was high among the girl students, but practice for proper menstrual hygiene was low comparted to studies done in similar settings. Still most of them were not aware of the hygienic practices to avoid infection, odour and frequency to change the sanitary pads.

## References

[1] Abioye-Kuteyi EA (2000). Menstrual knowledge and practices amongst secondary school girls in lle lfe, Nigeria. J R Soc Promot Health.

[2] Baisley K, Changalucha J, Weiss HA, Mugeye K, Everett D, Hambleton I (2009). Bacterial vaginosis in female facility workers in north-western Tanzania: prevalence and risk factors. Sex Transm Infect.

[3] Aniebue UU, Aniebue PN, Nwankwo TO (2009). The impact of pre-menarcheal training on menstrual practices and hygiene of Nigerian school girls. Pan Afr Med J.

[4] Ali TS, Rizvi SN (2010). Menstrual knowledge and practices of female adolescents in urban Karachi, Pakistan. J Adolesc.

[5] Mahon T, Fernandes M (2010). Menstrual hygiene in South Asia: a neglected issue for WASH (water, sanitation and hygiene) programmes. Gender and Development.

[6] Tiwari H, Oza UN, Tiwari R (2006). Knowledge, attitudes and beliefs about menarche of adolescent girls in Anand district, Gujarat. East Mediterr Health J.

[7] Adhikari P, Kadel B, Dhungel SI, Mandal A (2007). Knowledge and practice regarding menstrual hygiene in rural adolescent girls of Nepal. Kathmandu Univ Med J.

[8] Dhingra R, Kumar A, Kour M (2009). Knowledge and practices related to menstruation among tribal (Gujjar) adolescent girls. Studies on Ethno-Medicine.

[9] Adinma ED, Adinma JI (2008). Perceptions and practices on menstruation amongst Nigerian secondary school girls. Afr J Reprod Health.

[10] Santra S (2017). Assessment of knowledge regarding menstruation and practices related to maintenance of menstrual hygiene among the women of reproductive age group in a slum of Kolkata, West Bengal, India. Int J Community Med Public Health.

[11] Dasgupta A, Sarkar M (2008). Menstrual hygiene: how hygienic is the adolescent girl?. Indian J Community Med.

